# Supplementary figures and images for: Expression Analysis of MaTGA8 Transcription Factor in Banana and Its Defence Functional Analysis by Overexpression in Arabidopsis
Source: Int J Mol Sci. 2021 Aug 28;22(17):9344. doi: 10.3390/ijms22179344 (PMC8430518; doi:10.3390/ijms22179344)

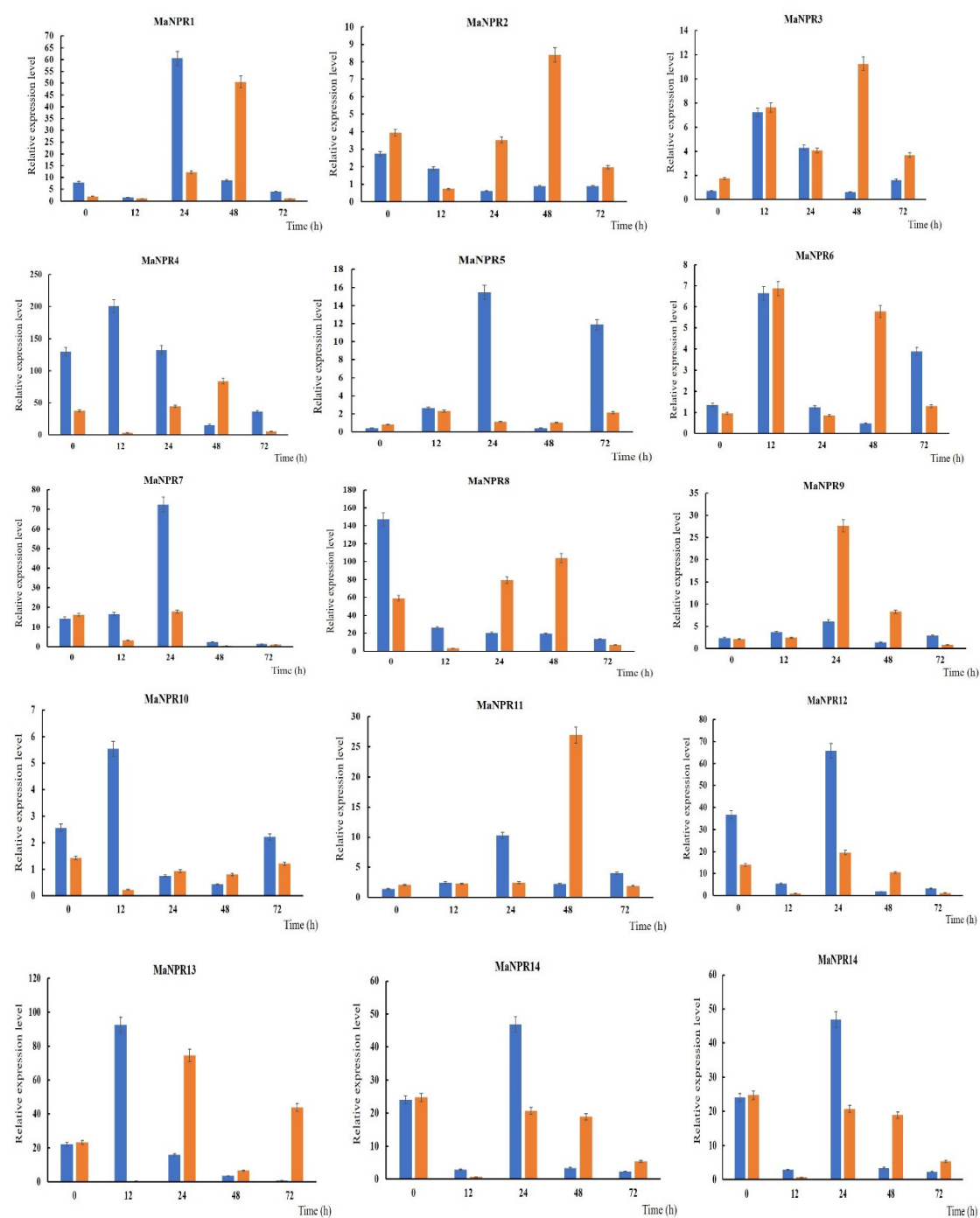

**Figure S1.** The relative expression of MaNPR1 family members under SA treatment.

Supplement: Supplementary file 1 [file ijms-22-09344-s001.zip › Supplementary_Material.pdf]
